# Primary Osteosarcoma of the Distal Fibula treated with Distal Fibulectomy with a Five-Year Follow-up: A Case Report

**DOI:** 10.5704/MOJ.1707.010

**Published:** 2017-07

**Authors:** I Saadon, B Amit, A Zolquarnian, F Muhamad

**Affiliations:** Department of Orthopaedics, Hospital Sultan Ismail, Johor Bahru, Malaysia; ^*^Newcastle University Medicine Malaysia, Iskandar Puteri, Malaysia

**Keywords:** distal fibula, osteosarcoma, limb salvage surgery

## Abstract

Musculoskeletal tumours of the lower limbs especially malignant tumours are not common. The fibula is the site of primary bone tumours as reported in 2.4% of lower limb tumours with the proximal third being more frequently involved than the distal segment. Osteosarcoma is the most common primary malignant bone tumour of nonhaematopoietic origin, with distal fibular involvement in 0.47% of patients. The advances in imaging techniques and neo-adjuvant chemotherapy have now made it possible to accurately define the extent of tumour and plan limb salvage with tumour resection. The purpose of this case report is to highlight the successful outcome of limb salvage procedure with a five year follow up in an 11-year old boy with distal fibular osteosarcoma. Limb salvage surgery with distal fibulectomy and retention of the foot are a good alternative to radical amputation.

## Introduction

Musculoskeletal tumours of the lower limbs especially malignant tumours are not common. They are considered to exhibit lower mortality rates as compared to other sites. Synovial cell sarcoma, osteosarcoma and Ewing’s sarcoma are reported as the more common mesenchymal cancers of the lower limbs. The fibula is the site of primary bone tumours as reported in 2.4%, the proximal third being more frequently involved than the distal segment. It has been observed that malignancies involving the distal third of the fibula carry a better prognosis than proximal lesions. Osteosarcoma is the most common primary malignant bone tumour of nonhaematopoietic origin with the distal fibular localizations in 0.47 % of patients^1^. This is a rare presentation and the management presents unique challenges.

The natural history of aggressiveness of primary osteosarcomas has led to the consideration of amputation as the choice for local control of the tumour in the limb. With the advances in imaging techniques and neo-adjuvant chemotherapy, it is now possible to accurately define the extent and therefore plan limb salvage with tumour resection. However, the wider resections of the entire distal fibula can result in potential loss of ankle stability and therefore the need for reconstruction of a stable ankle joint and sufficient skin coverage of the area. Furthermore, the incidence of inadequate excision margins has been reported to be higher after limb-salvage surgery for fibular osteosarcomas compared with other appendicular lesions that are responsible for poor outcomes related to high rate of local recurrence and metastasis. Recent literature has challenged this view and reported no recurrence and no impact on survival in patients with fibular osteosarcomas who had undergone intentional marginal resection followed by adjuvant therapy^2^. The advances in imaging techniques and adjuvant therapy have allowed the surgeon to accurately define the extent and therefore plan tumour resection rendering limb-salvage surgery as a feasible option. The purpose of this case report is to record the successful outcome of limb salvage procedure at five year follow up in an 11 year-old boy with distal fibular osteosarcoma.

## Case Report

**Fig. 1:**
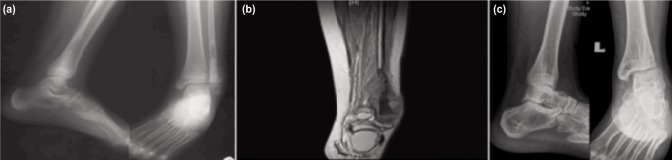
(a) Expansile lytic lesion involving the distal third of fibula with epiphysis with some patchy sclerosis and cortical breach, (b) MRI of left fibula showing mass lesion within and encircling the distal fibula with proximal extension of the marrow and cortex with central necrosis and (c) Post-surgical follow up; Radiograph of ankle joint shows valgus and talar shift with no signs of degeneration in last follow up in 20 December 2016.

An 11 year-old boy was first seen with the chief compliant of pain and swelling at the lateral aspect of the left ankle of two months’ duration. There was no history of injury, fever, loss of appetite or weight loss. Local examination revealed an illdefined swelling of 5cm x 4cm at the lateral part of left ankle which was warm and tender to touch. There was terminal restriction of movement at the ankle joint with no evidence of distal neuro-vascular deficit. Radiographs revealed an expansile lytic lesion involving the distal third of fibula with epiphysis with some patchy sclerosis and cortical breach. The proximal extension of the marrow and cortex is about 22.5cm from the tip of the fibula (Fig. 1a).

**Fig. 2:**
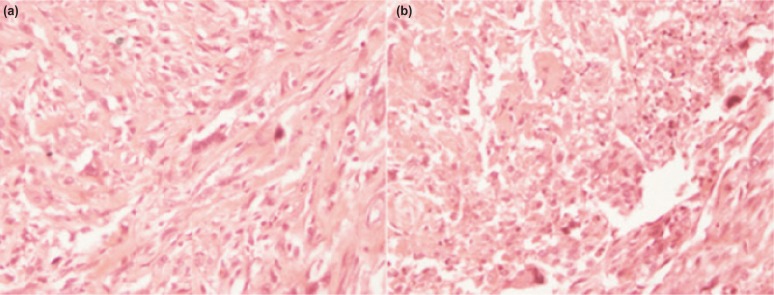
(a) High power microscopic image of osteogenic sarcoma demonstrating tumour cell surrounded by haphazardly arranged dense pink matrix with high pleomorphism and (b) High power microscopic image of osteogenic sarcoma with area of tumour necrosis.

MRI showed an aggressive lesion involving the distal third of the right fibula with involvement of distal epiphysis, and with cortical breach and soft tissue extension (Fig. 1b). Chest CT scan revealed no evidence of metastasis. A diagnosis of malignant neoplastic lesion of distal fibula was made. Core biopsy revealed histopathological features of osteosarcoma with tumour cells demonstrating high pleomorphic and many nuclei and prominent nucleolus. The tumour cells were surrounded by haphazardly arranged dense pink matrix. This was typical osteoid produced by malignant tumour cell of osteosarcoma. (Fig. 2a, 2b). A decision to perform wide fibular resection was made as tumour was localized to lateral compartment with no metastases. Three courses of neoadjuvant chemotherapy were given at three week intervals. Post chemotherapy MRI revealed cortex and marrow involvement of the left fibula measuring 10.7 cm from the lateral malleolus.

**Fig. 3:**
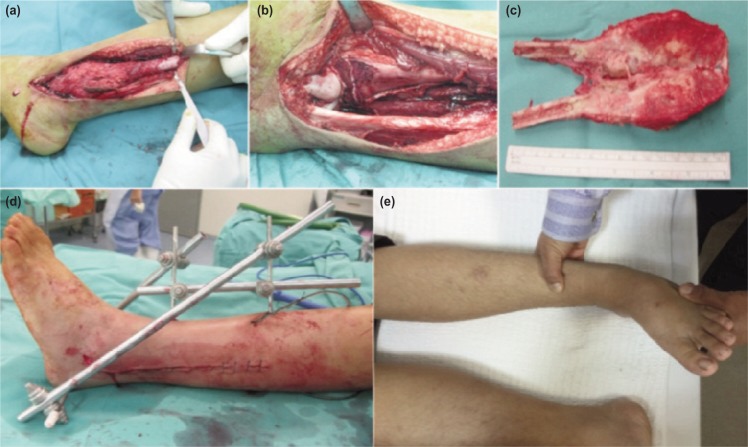
(a) Intraoperative photographs demonstrating the tumour mass arising from the distal fibula (b) Clinical figure after removing tumour tissue, (c) Excised tumour specimen for histopathological examination, (d) Post op clinical figure demonstrating adequate soft tissue coverage with external fixator and (e) Follow up clinical figure demonstrating varus stress test.

Patient underwent distal fibulectomy through a direct lateral longitudinal incision. At operation, the lesion was about 11 cm in diameter with involvement of flexor hallucis longus, peroneus brevis and the peroneal artery (Fig. 3a). About 15 cm of fibula was resected above the tip of the lateral malleolus and marginal excisions of flexor hallucis longus and peroneus brevis were carried out leaving behind the distal tendons intact (Fig. 3b). Peroneal artery was ligated. Ankle reconstruction was done using soft tissue and tested clinically for stability. Haemostasis was achieved and the wound closed in layers. External fixator was applied and sterile dressing done (Fig. 3d).

Sutures were removed after two weeks and patient was mobilised with non-weight axillary crutches. At 12 weeks, the external fixator was removed and fibreglass cast was applied and full weight bearing started. Post-operatively, chemotherapy with Doxorubicin and Cisplatin was administered every three months for four cycles. Patient was followed up at intervals of three weeks for the first three months, three monthly for one year, six monthly for two years and every year for five years. During his last follow up in December 2016, there is no pain or swelling and the ankle joint was stable. Radiograph showed valgus and talar shift but no sign of joint degeneration. (Fig. 1c) However, the varus and valgus stress tests at ankle was negative (Fig. 3e). He was able to participate in regular sports including running, cycling and playing basketball.

## Discussion

The 1970’s witnessed the management of high grade osteosarcomas solely with amputation. This was attributed to the limitations in the imaging modalities that precluded accurate assessment of the real size of the tumour and its extension beyond the cortices. There is recent evidence in literature supporting limb salvage procedures as the main stay of treatment for high grade osteosarcomas. However, the wide resections of the entire distal fibula results in loss of ankle stability and inadequate soft tissue conditions such as insufficient skin coverage of the area^3^. This mandates reconstruction of a stable ankle joint and the need for skin grafting or flap.

A PubMed literature review on the surgical treatment of distal fibular tumours showed paucity of results with only case reports and small case series. This is related to the rarity of distal fibular tumours. A review by Capanna *et al* reported the outcome in 11 patients who underwent distal fibular resection for benign or malignant tumours with limb salvage surgery and different techniques of reconstruction. In this case series, seven patients recovered normal function, while four had reduced mobility. One patient developed lateral subluxation of the talus. However, all patients were free of pain and the functional and clinical outcomes were independent of the nature of the lesion^2^.

Various surgical techniques for different distal fibular pathologies have been proposed. Recent advances in surgical techniques, chemotherapy and bioengineering technology for mechanical prosthesis have led to the introduction of alternatives to amputation. One of the frequently performed technique is distal fibular resection without reconstruction of the lateral side of the ankle. In these situations, ankle stability is obtained by either soft tissue and ligament reconstruction or tibiotalar arthrodesis. If reconstruction is followed after resection, the choice is using allografts, autografts or pedicled vascularised epiphyseal transfers using the ipsilateral proximal fibula. A prosthetic ankle joint replacement is an option^4^.

All these surgical techniques however pose common complications such as varus instability, valgus collapse and limited ROM. Furthermore, the unique associated complications include donor site morbidity, lateral knee instability, peroneal nerve damage, incongruity between the articular surfaces of the ankle joint, and non-union with reconstruction with fibular head and occurrence of talar collapse which can occur after ankle joint replacement^5^. However there are no specific guiding principles in the choice of the technique and there is need for long term studies and randomised controlled trials to develop protocols on this subject.

With the given evidence, the authors conclude that sufficient evidence has emerged for limb salving surgeries for distal fibular tumours including the rare primary osteosarcoma with improved quality of life. Distal fibular osteosarcoma carries a good prognosis if sound oncological margins can be achieved during the initial surgery. In the presence of the malignant tumour not involving the lateral malleolus and located at least 2 cm proximal to the tibiotalar joint, meta-diaphyseal fibular resection and periosteal tibial stripping is performed, followed by arthrodesis between the remaining lateral malleolus and the distal tibia described as Capanna’s technique^2^. In the presence of the malignant tumour involving the lateral malleolus, fibular resection with or without reconstruction or prosthetic ankle replacement may be recommended. The lateral malleolus can be removed with remarkably little functional deficit. Post-operative chemotherapy plays an important role in the management of osteosarcoma to prevent micro-metastasis and local tumour recurrence with improved disease-free survival rates.

## Conflict of Interest

The authors declare no conflicts of interest in the preparation of the manuscript.
